# From wild-type to Omicron: changes in SARS-CoV-2 hospital cluster dynamics. Observations from a German tertiary care hospital

**DOI:** 10.3205/dgkh000474

**Published:** 2024-04-17

**Authors:** Britta Kohlmorgen, Annika Brodzinski, Sandra Jendrossek, Thorsten Jeske, Anne-Kathrin Putsch, Maja Weisker, Sandra Schneider, Frank Schwab, Petra Gastmeier, Sonja Hansen

**Affiliations:** 1 Charité – Universitätsmedizin Berlin, corporate member of Freie Universität Berlin and Humboldt-Universität zu Berlin, Institute of Hygiene and Environmental Medicine, Berlin, Germany; 2 DRK Kliniken Berlin, Institute for Hygiene, Berlin, Germany; 3 Evangelisches Waldkrankenhaus Spandau, Berlin, Germany; 4 Helios Klinikum Emil von Behring, Berlin, Germany

**Keywords:** SARS-COV-2, COVID-19, nosocomial infection, hospital-acquired

## Abstract

**Aim:**

SARS-CoV-2 hospital clusters are a challenge for healthcare systems. There is an increased risk of infection for both healthcare workers (HCWs) and patients; cluster countermeasures are also a drain on resources for the wards affected. We analysed to which extent characteristics and dynamics of SARS-CoV-2 clusters varied throughout the pandemic at a German university hospital.

**Methods:**

Patient and/or HCW clusters from 10/2020 to 04/2022 were included in the study and grouped by virus variant into i.) clusters comprised of the presumably predominant wild-type, Alpha or Delta (WAD) SARS-COV-2 variants, and ii.) clusters comprised predominantly of Omicron subtype cases. The two groups were compared for specific characteristics and dynamics.

**Results:**

Forty-two SARS-CoV-2 clusters and 528 cases were analysed. Twenty-one clusters and 297 cases were attributed to the WAD and 21 clusters and 231 cases to the Omicron group. There were no significant differences in median size (8 vs. 8 cases, p=0.94) or median duration (14 vs. 12 days; p=0.48), nor in the percentage of HCWs involved (46.8% vs. 50.2%; p=0.48). Patients in the WAD group were older (median 75 vs. 68 years of age; p≤0.05). The median time from cluster onset to case onset was significantly shorter for the Omicron group (median 6 vs. 11 days; p≤0.05).

**Conclusions:**

Omicron clusters exhibited a more rapid dynamic, forcing all parties involved to adapt to the increased workload. Compared to excessive community case counts, constant Omicron cluster-affiliated case counts and stable cluster characteristics suggest an improved compliance with IPC countermeasures.

## Zusammenfassung

**Zielsetzung:**

Cluster bzw. Häufungen von SARS-CoV-2 Infektionen in Krankenhäusern stellen weiterhin eine Herausforderung für das Gesundheitssystem dar. Zum einen ergibt sich aus den Häufungen ein erhöhtes Infektionsrisiko für Patien:innen und Mitarbeiter:innen, zum anderen sind die zu ergreifenden Gegenmaßnahmen Ressourcen verzehrend für die betroffenen Abteilungen. Nachfolgend stellen wir eine Analyse des Ausmaßes des Wandels von Cluster Charakteristika und Cluster Dynamik im Zuge der ersten beiden Pandemiejahre in einem deutschen Universitätsklinikum dar.

**Methoden:**

Patient:innen und/oder Mitarbeiter:innen Cluster von 10/2020 to 04/2022 wurden in die Analyse integriert und entsprechend der zugrunde gelegten Virusvariante eingeteilt in i.) Cluster mit vorwiegend Wildtyp, Alpha oder Delta (WAD) SARS-COV-2 Fällen und ii.) Cluster mit überwiegend Omikron Fällen. Beide Gruppen wurden in Bezug auf spezifische Cluster Charakteristika und ihre Cluster Dynamik miteinander verglichen.

**Ergebnisse:**

42 SARS-CoV-2 Cluster und 528 Fälle wurden analysiert. 21 Cluster und 297 Fälle wurden zu der WAD Gruppe und 21 Cluster und 231 Fälle zu der Omikron Gruppe gezählt. Es fanden sich keine signifikanten Unterschiede in der medianen Clustergröße (8 vs. 8 Fälle, p=0.94) oder der medianen Dauer (14 vs. 12 d; p=0.48) oder im Anteil der beteiligten Mitarbeiter:innen (46.8% vs. 50.2%; p=0.48). Die Patient:innen der WAD Gruppe waren insgesamt älter (Median 75 vs. 68 Jahre; p≤0.05). Die Zeit zwischen Clusterbeginn und dem Zeitpunkt, zudem im Median ein Fall detektiert wurde, war in der Omikron Gruppe signifikant kürzer (6 vs. 11 d; p≤0.05).

**Schlussfolgerungen:**

Die Omikron Cluster zeigten eine verdichtete Dynamik, die für alle Beteiligten eine Anpassung an die anfallende Arbeitslast notwendig machte. Im Vergleich zu der exzessiv ansteigenden Inzidenz außerhalb der Klinken blieben die Omikron zugeordneten Cluster stabil in Bezug auf Dauer, Größe und Mitarbeiterbeteiligung, was auf eine verbesserte Adhärenz zu den Cluster-Gegenmaßnahmen hinweist.

## Introduction

One of many SARS-CoV-2 pandemic-related challenges for healthcare systems are COVID-19 clusters in healthcare facilities, such as hospitals or long-term care facilities [1], [2], [3], [4]. Since COVID-19 is predominantly an airborne disease [5], [6], [7] and infectivity is possible before the onset of symptoms [8], [9], its containment within facilities is often troublesome. In particular, cases among unidentified healthcare workers (HCWs) or patients can lead to minor or major clusters [10], [11]. HCWs as an essential group and patients as a vulnerable group are at higher risk of exposure, infection, and/or adverse clinical outcomes from SARS-CoV-2. Furthermore, hospital cluster countermeasures often include capacity-reducing actions, e.g., closure of wards/facilities or quarantine and isolation of HCWs.

In addition to the wild-type SARS-CoV-2 strain, various virus variants such as Alpha, Delta, or Omicron have emerged over the course of the pandemic. With changes in which virus variant is predominant, infection dynamics have also varied over time [12], [13], [14]. In particular, infections from Omicron subtypes differ from the wild-type to Delta subtype (hereinafter referred to as WAD) infections, since Omicron has a shorter incubation period, is more contagious; moreover, vaccination efficacy is lower [13], [15].

Studies thus far have been single cluster-analyses [16], [17], [18], [19] or have described structural data for cluster-related cases at a national level, e.g., in Germany or the UK [20], [21], [22]. Here, we present our experiences of the SARS-CoV-2 pandemic in a tertiary care university hospital and compare the characteristics and dynamics of WAD and Omicron clusters. 

## Methods

### Study type and setting

In this retrospective cluster analysis, we included data from October 2020 to April 2022. All clusters were detected in an 890-bed hospital belonging to a large urban university centre located in eastern Germany. Notably, the clinic does not provide paediatric, gynaecologic or obstetric care. The infection prevention and control (IPC) team, responsible for cluster detection, consisted of up to 7 members, 4 of them physicians.

### Cluster definition and identification

To be included in this analysis, a cluster had to have at least ONE of the following:

1. one HCW and one (potentially) nosocomial patient case;
2. two (potentially) nosocomial patient cases;
3. three HCW cases.

The IPC team evaluated the epidemiological linkage be-tween cases, usually without sequencing information, since sequencing capacity was limited. To be considered a potentially nosocomial case, a patient had to have been admitted to the ward/department affected by the cluster at least three days or more prior to a positive SARS-CoV-2 test or the onset of symptoms. Possible index cases with an onset before day three of their hospital stay, and who were evaluated as cluster affiliated, were also included. Clusters in departments that were not related to patient care were excluded, as were clusters on wards exclusively treating COVID-19 patients. 

Identification of a cluster could result from either (a) SARS-CoV-2 screening measures on wards with (potential) nosocomial COVID-19 cases); (b) a report from HCWs of a suspected link between cases on the ward; (c) a report from the department of occupational medicine of more than one HCW case; or (d) a notification by the semi-automated cluster detection tool (CLAR) used by the IPC team [23]. 

### Countermeasures for SARS-CoV-2 pandemic in general and specific measures for SARS-CoV-2 clusters

**Figure 1:**
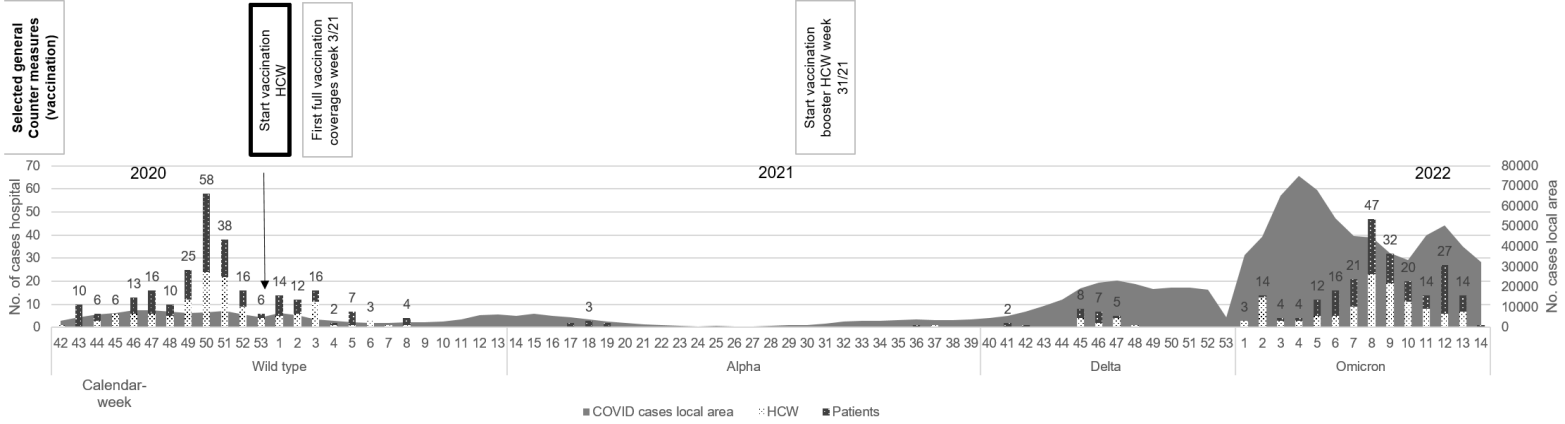
Longitudinal description of cluster-affiliated COVID-19 cases in the hospital in comparison to reported COVID-19 cases in the region including selected general SARS-CoV-2 (vaccination) countermeasures or adapted measures

General pandemic countermeasures included the HCW vaccination program, PCR screenings of patients upon admission and throughout their stay, regular rapid antigen testing for HCWs, the adjustment of personal protective equipment, training of donning and doffing procedures, isolation and quarantine procedures for HCWs and patients, and instructions for hospital visitors. Most of these measures were implemented before the beginning of the time span analysed, but were adapted to the changing availability of resources (e.g., personal protective equipment, PCR and antigen tests, the HCW vaccination campaign) and epidemiological requirements throughout the pandemic. A selection of these measures with emphasis on the HCW vaccination campaign are shown in Figure 1 (Fig. 1). Standard cluster countermeasures were established early in the pandemic and have been modified to meet the most current epidemiological or regulatory needs (Table 1 (Tab. 1)).

### Data collection and analysis

Patient data was obtained from electronic files and was provided by the relevant department. HCW data was provided by the relevant department and by the department for occupational medicine. In most affiliated HCW cases, a telephone interview was performed by the IPC team to confirm the epidemiological linkage and to evaluate possible (close) contacts. 

To compare WAD- and Omicron-related clusters, we divided all clusters and associated cases into two groups: (1) those predominantly with WAD variants and (2) those with predominantly with the Omicron variant. Since sequencing capacities were restricted, cluster affiliation with either group was based on the predominant (>90%) local variant at the time of cluster detection. The switch from Delta to Omicron variants took place in the hospital’s area at the beginning of January 2022. There were no clusters detected during the transition between variant groups. The two groups were compared for specific characteristics, e.g., duration, cluster size, affiliation with a particular occupational group (physicians, nursing staff or others, such as physiotherapists, cleaning personnel, etc.) together with their cumulative epidemic curves.

The cumulative epidemic curves show the “days to positivity” for each case related to the beginning of the cluster. This was calculated by subtracting the date of the positive SARS-CoV-2 test of each individual case from the date of onset of the related cluster. All cases with a presumed onset on a particular cluster day were cumulated separately for WAD and Omicron groups, starting at day 0, and presented graphically. 

Cluster duration, whenever used for descriptions, was defined as the difference (in days) between the laboratory-confirmed onset date of its first and last affiliated SARS-CoV-2 case. European standards were used for calendar-week calculations [24]. Statistical data from the surrounding region was derived from the German SurvStat [25] database operated by the Robert Koch Institute. 

### Statistical analysis

The univarable comparison of discrete data was performed with chi-squared tests and that of continuous data with Wilcoxon rank-sum tests. Significance was set at p<0.05. Both tests were performed with R studio software, version 1.2.5001. All graphics and tables were created with Microsoft Excel 2016. The analysis was exploratory in nature.

### Ethical considerations

All data used in this analysis derived from surveillance data on healthcare-associated infections and from clusters reported to healthcare authorities, in accordance with the German Protection Against Infection Act [26]. We present only aggregated and anonymised secondary data. Therefore, no further ethical approval or informed consent was obtained. 

## Results

In total, we identified 42 SARS-CoV-2 clusters with 528 affiliated cases over 68 weeks of observation, from 17 October 2020 to 06 April 2022. During the WAD phase, 297 cluster-affiliated cases were identified; 231 cases were identified during the Omicron phase. In the 57 weeks of observation during the WAD period, there were 38 weeks with active clusters (66.7%). All of the 11 weeks of observation during the Omicron period contained clusters. The cluster characteristics determined (Table 2 (Tab. 2)) show no significant differences between the WAD and Omicron groups in terms of median cluster size (8 vs. 8 cases), duration (14 vs. 12 days), age of patients or HCW in all cases (55.5 vs. 52 years), HCW age (36.5 vs. 36), or the percentage of physicians (12.8% vs. 16.5%) and nursing staff (21.2% vs. 26.8%). Significant differences between the two groups can be seen in the percentage of HCWs, other than physicians or nursing staff, the median patient age (75 vs. 68 years), and the median days to positivity (duration between cluster and individual case onset) (11 vs. 6 days). 

Two major peaks in cluster-affiliated cases can be seen during the WAD and the Omicron periods, with but little latency to the peak of community case counts (Figure 1 (Fig. 1)). Both peaks in cluster-affiliated cases reach an almost equal maximum of 58 cases during the WAD phase (week 50/2020) and maximum of 47 cases during the Omicron phase (week 8/22). In the community, the WAD case-count peaks were lower than Omicron’s, with the maxima reached in week 46 of 2020 (n=8,456) of the wild-type phase, in week 47 of 2021 (n=23,385) of the Delta phase, and in week 12 of 2022 with 50,365 cases during the Omicron phase.

**Figure 2:**
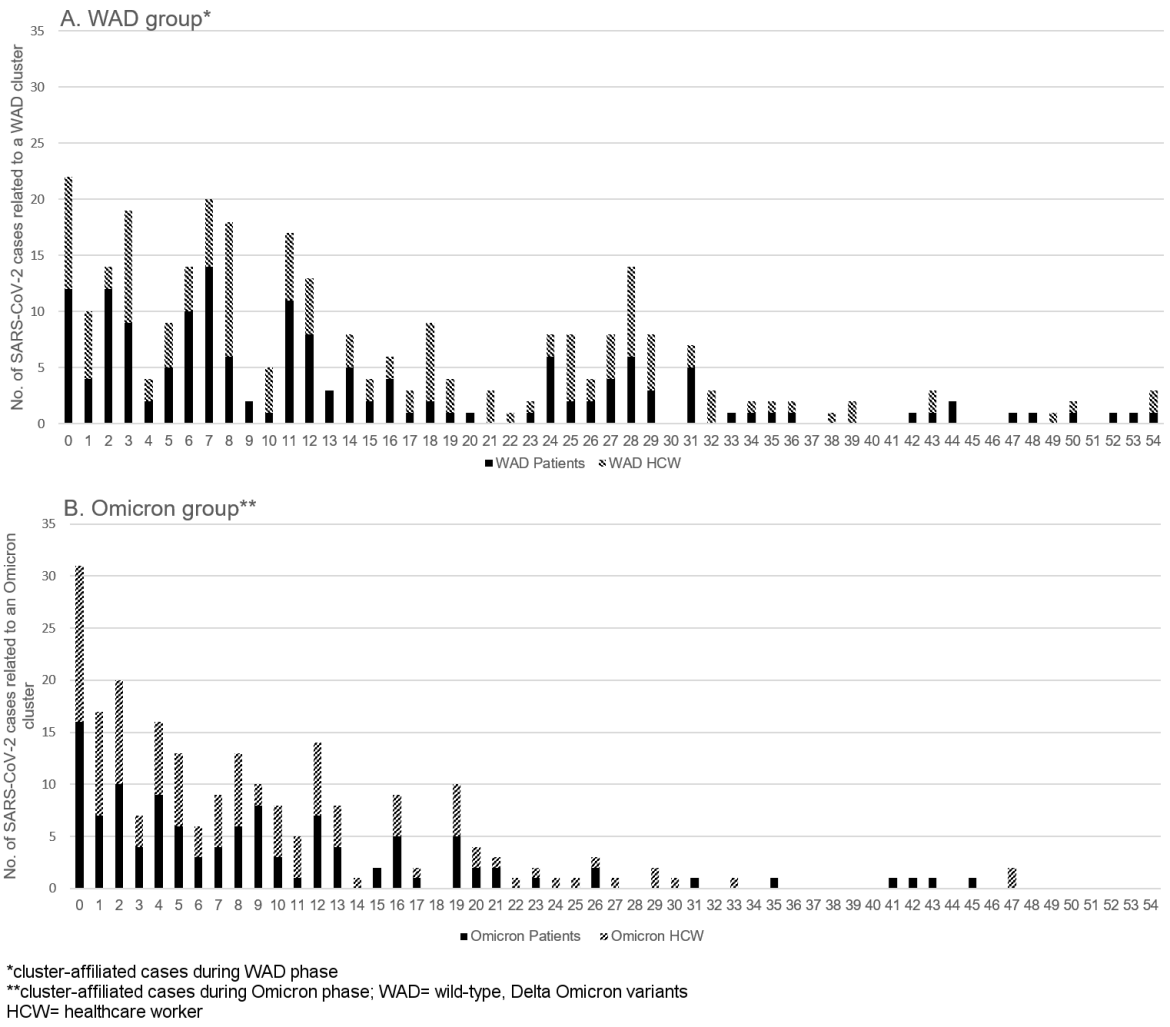
Cumulative epidemic curves of cluster-affiliated cases.y-axis: no. of cases, x-axis: days to positivity for each case (difference between cluster onset and case onset)

The cumulative epidemic curves show aspects of WAD and Omicron cluster dynamics (Figure 2 (Fig. 2)). For the WAD period, the distribution of cluster-affiliated COVID-19 cases stretches broadly from day 0 to day 54 of the clusters. Multiple peaks in case counts are visible during this period, e.g., on cluster days 0, 3, 7, 1, 18 and 28. Cluster-related COVID-19 cases during the Omicron period were detected until day 47 of the clusters. The Omicron curve is compressed, and only a few peaks are clearly discernable (Figure 2 (Fig. 2)).

## Discussion

SARS CoV-2 infection dynamics varied during the pandemic, depending on the predominant virus variant then circulating. Notably, most WAD cases occurred during the time when the wild-type variant was predominant and the vaccination program had not yet started. Therefore, the cluster-affiliated workload was highest when the wild-type variant encountered an unvaccinated population and after the predominant SARS-CoV-2 variant had been replaced by Omicron. Although the WAD period observed was 5 times longer than the Omicron phase (3 times longer for active weeks only), 50% of all clusters and 44% of all cases had to be managed in the shorter Omicron phase. This higher cluster density might be a reflection of the extremely elevated community case counts during the Omicron phase. Nonetheless, it did not lead to significant differences between WAD and Omicron clusters in regard to median size, median duration, or in the percentage of affected HCWs or patients. This could be indicative of more effective cluster containment – e.g., as a result of training – and/or altered cluster dynamics which favored this effect. 

In addition to the aforementioned higher cluster density during the Omicron phase, we were able to show a more rapid Omicron cluster dynamic in the cumulative epidemic curves. The median time span to positivity (duration from the onset of a cluster until the onset of each individual case) is significantly shorter for Omicron clusters, rendering the cumulative epidemic curve of Omicron cluster-affiliated cases more compact, showing fewer distinguishable peaks than the broader curve of WAD cluster-affiliated cases. This could be either an expression of the shorter median incubation period of infections with the Omicron variant, fewer secondary cluster cases during that phase, or a combination of both. The changed dynamic led to an increased workload for the wards and the IPC team on the one hand, but might have also led to a quicker termination of clusters on the other.

In addition to these observations, two notable characteristics which are significantly different for each group point to weaknesses of this study. First, the patient age was significantly lower for Omicron cluster-affiliated cases. This might be a manifestation of the success of the public vaccination program, which targeted elderly and vulnerable groups, especially at the beginning of the pandemic. Other effects or implications on cluster dynamics are also possible. For example, compliance with countermeasures, such as isolation or quarantine procedures and/or mobility of patients might differ between groups. Given the relatively small set of characteristics in the univariable analysis, other unknown differences between the groups might also have had an impact on cluster dynamics. Second, significantly fewer personnel reported as “other staff” (i.e., HCWs who were neither physicians nor nursing staff) were identified in Omicron clusters. Skyrocketing community case counts during the Omicron phase made cluster attribution of HCWs more difficult, especially for non-stationary HCWs, such as physiotherapists, cleaning personnel, psychologists etc. This effect could have led to an underestimation of this group. 

A further limitation of this study was the limited capacity for sequencing. Therefore, the majority of viral strains were not compared genotypically for cluster or variant group affiliation. Since all clusters and their epidemiological background were evaluated by the IPC team and reported to the health authorities, this work concerns IPC routines and the community distribution of virus variants. However, it is possible that the number of clusters was overestimated. 

In conclusion, cluster dynamics assumed a more rapid pace between the early and later COVID-19 pandemic phases, with negative consequences for workload and countermeasure resource availability. However, constant improvement and an increase in experience with general outbreak measures (e.g., vaccination, PPE) as well as specific cluster management measures (e.g., containment procedures, contact tracing, etc.) counteracted these negative effects.

## Notes

### Competing interests

The authors declare that they have no competing interests.

## Figures and Tables

**Table 1:**
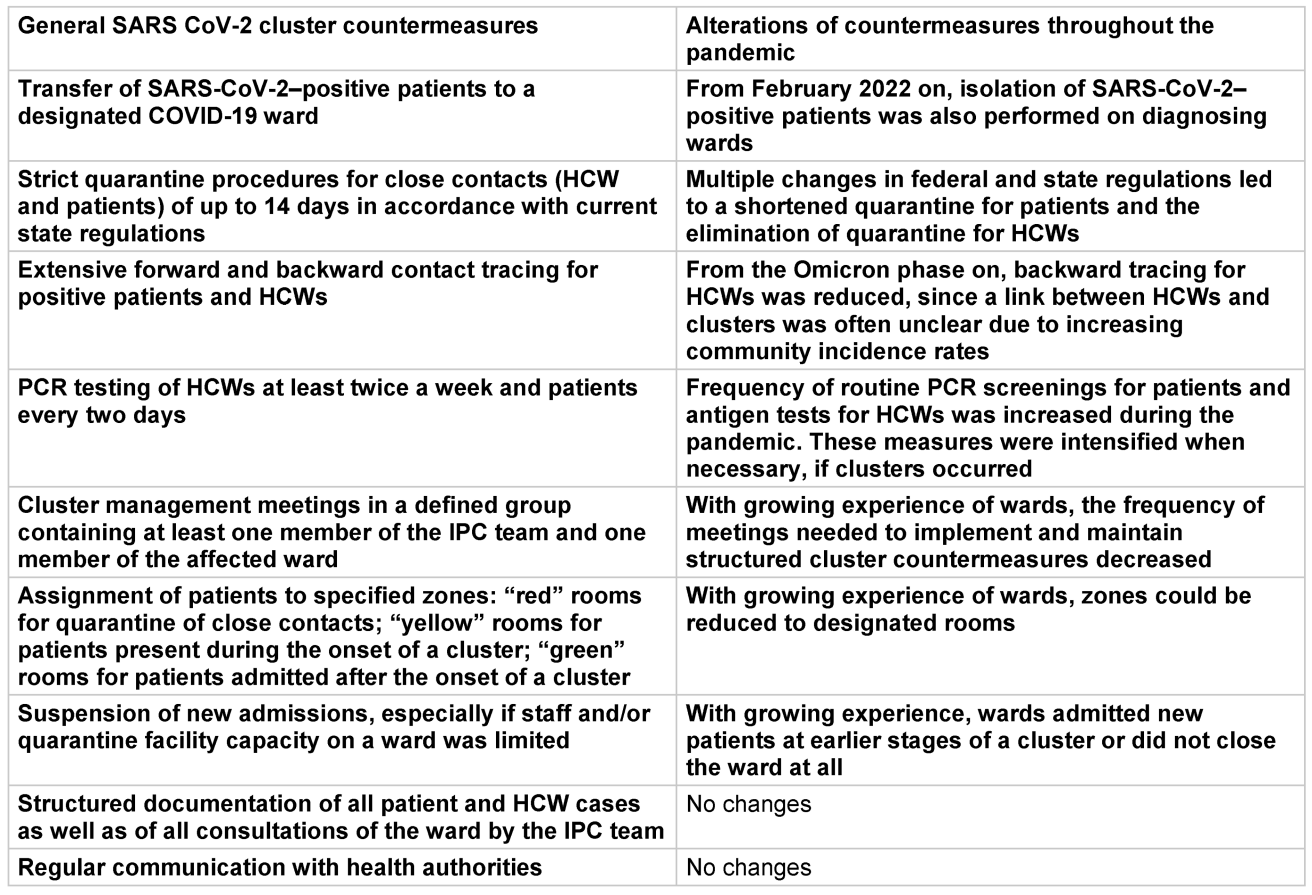
Specific cluster countermeasures throughout the course of the pandemic in the analysed hospital

**Table 2:**
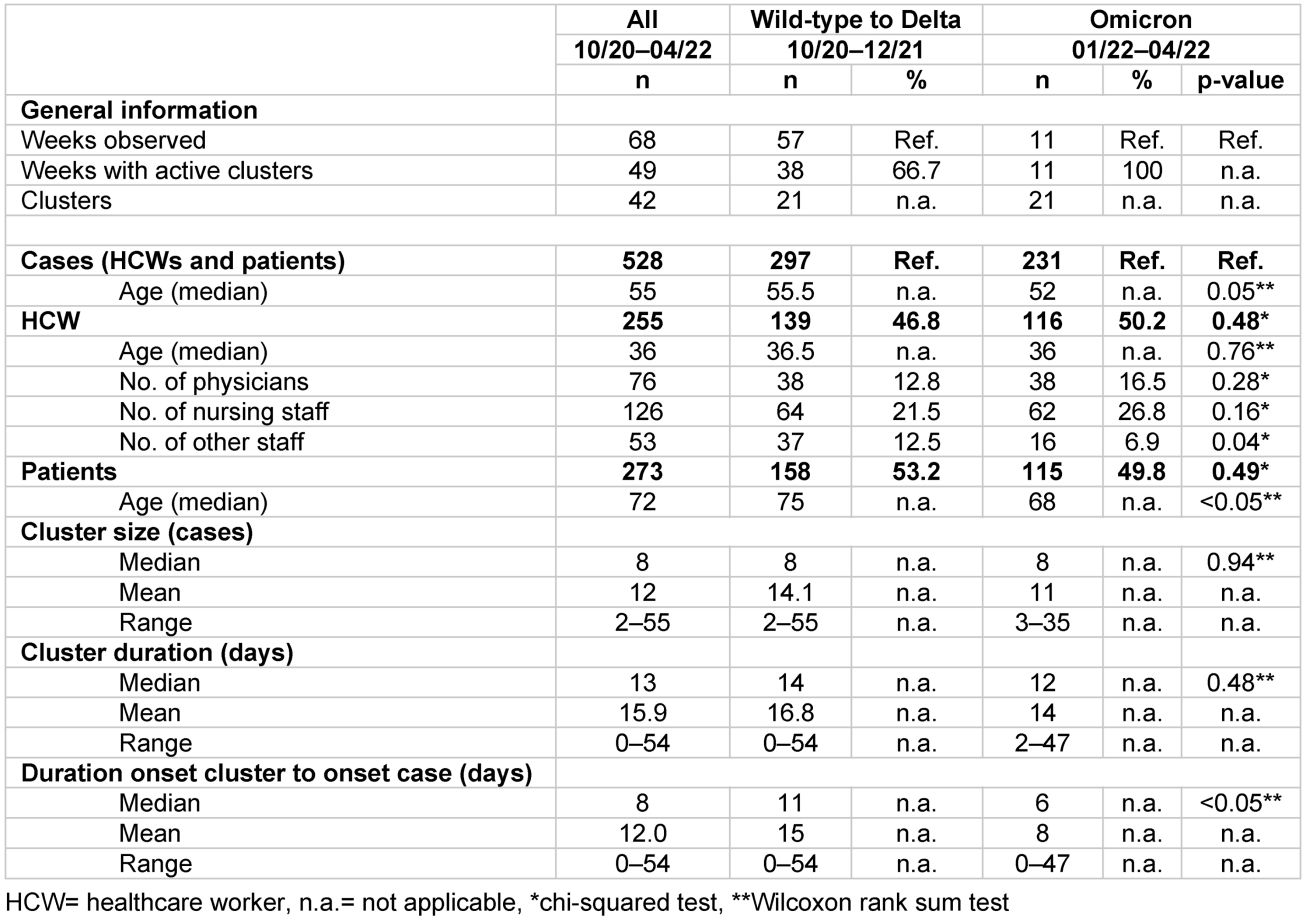
Description of clusters and cluster-affiliated cases. Comparison of wild-type to Delta (WAD) and Omicron cluster characteristics with p-values where applicable.
